# A Curcumin-BODIPY Dyad and Its Silica Hybrid as NIR Bioimaging Probes

**DOI:** 10.3390/ijms23179542

**Published:** 2022-08-23

**Authors:** Chiara Maria Antonietta Gangemi, Tania Maria Grazia Salerno, Anna Barattucci, Fabio Cucinotta, Paola Bonaccorsi, Giovanna Calabrese, Paola Poma, Maria Giovanna Rizzo, Sebastiano Campagna, Fausto Puntoriero

**Affiliations:** 1 Dipartimento di Scienze Chimiche Biologiche Farmaceutiche ed Ambientali (CHIBIOFARAM), Università degli Studi di Messina, 98166 Messina, Italy; 2 School of Natural and Environmental Sciences, Newcastle University, Newcastle upon Tyne NE1 7RU, UK; 3 Dipartimento di Scienze e Tecnologie Biologiche, Chimiche e Farmaceutiche (STEBICEF), Università degli Studi di Palermo, 90128 Palermo, Italy

**Keywords:** luminescence, curcumin, BODIPY, NIR probes, bioimaging, bichromophoric dyad

## Abstract

In this paper we describe the synthesis of a novel bichromophoric system in which an efficient photoinduced intercomponent energy transfer process is active. The dyad consists of one subunit of curcumin and one of BODIPY and is able to emit in the far-red region, offering a large Stokes shift, capable of limiting light scattering processes for applications in microscopy. The system has been encapsulated in MCM-41 nanoparticles with dimensions between 50 and 80 nm. Both the molecular dyad and individual subunits were tested with different cell lines to study their effective applicability in bioimaging. MCM-41 nanoparticles showed no reduction in cell viability, indicating their biocompatibility and bio-inertness and making them capable of delivering organic molecules even in aqueous-based formulations, avoiding the toxicity of organic solvents. Encapsulation in the porous silica structure directed the location of the bichromophoric system within cytoplasm, while the dyad alone stains the nucleus of the hFOB cell line.

## 1. Introduction

**Scheme 1:**
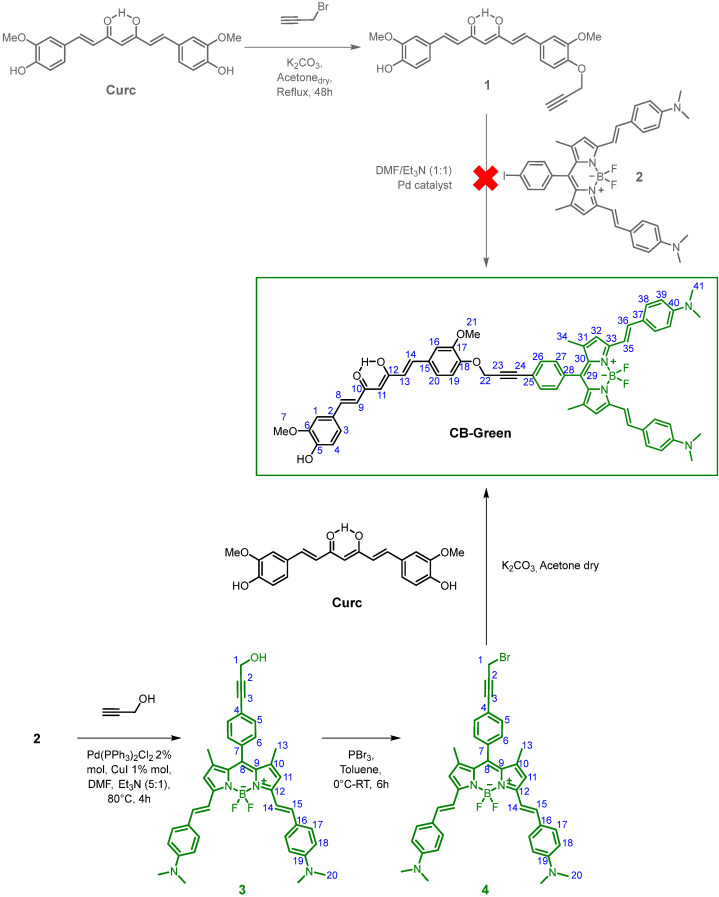
Synthetic route to **CB-Green** (**Curc:** curcumin; **CB-Green:** curcumin-BODIPY dyad).

In the past two decades, considerable progress has been made in the development of non-invasive and highly sensitive biomedical imaging techniques for clinical diagnosis and therapy assessment of pathological events associated with human diseases. The diversification of imaging modalities, such as X-ray, computed tomography (CT), magnetic resonance imaging (MRI), photoacoustic imaging (PI), positron emission tomography (PET) and fluorescence imaging (FI) has facilitated early diagnoses and targeted therapies. Compared to other well-developed techniques, FI with small-molecule dyes, in the near infrared region (NIR, 700–1000 nm), has been widely applied for real-time detection of biological species and identification of cancer cells or lymph nodes, as well as for the intraoperative image-guided surgical removal of pathological tissues, owing to its high sensitivity, excellent resolution, minimum photodamage to tissues, quick feedback, non-hazardous radiation and, last, but not least, its low costs and sustainability [1,2,3]. Most of the traditional fluorescent probes, such as rhodamine, cyanine, or coumarin derivatives, have absorption and emission in the UV-Vis range and the interference with haemoglobin, myoglobin, and other haemoproteins leads to light scattering and impaired tissue penetration. The natural emission of light by such biological structures, upon light absorption, generates a noise background that might even wash out the image of targets. For instance, curcumin (**Curc**), a yellow pigment, naturally obtained from the rhizomes of *Curcuma longa*, with a highly delocalized π-electron symmetric structure, is reported to possess good optical properties, with the advantage of being highly biocompatible [4,5]. Curcumin-based fluorescent probes can overcome the shortcomings of organic fluorescent dyes, such as poor lipophilicity and low quantum yield, as well as display high sensitivity and molecular target ability that avoid possible interference of other substances with similar structures. However, the inability to image in the NIR region, because of the short absorption wavelengths, ranging from 410 to 430 nm, as well as the poor chemical and photochemical stability, due to the fast decomposition at pH ≥ 7, and the severe losing of brightness upon photo-illumination, have limited their use as fluorescent dyes, especially regarding in vivo applications. Synthetic organic small-molecule NIR fluorophores are advantageous because they can be rationally tailored providing creativeness in structural design, low cytotoxicity and good cell permeability [6]. Furthermore, the hydrophobic and electrostatic interactions, along with hydrogen bonding between organic fluorescent dyes and biological species can lead to better sensitivity, selectivity, and bio-imaging capacity for diagnostics. For instance, 4,4-Difluoro-4-bora-3a,4a-diaza-s-indacene (BODIPY), and its derivatives, have received attention for their excellent photo and chemical stability, high molar absorption coefficients and fluorescence quantum yields. A wide variety of biocompatible BODIPY-based fluorescent dyes have been developed [7] and some of them are commercially available. The photophysical properties of BODIPY can be easily tuned by modification of its backbone. Its derivatives have been used for many biological applications [8,9,10,11,12]. One common strategy exploited to achieve red- or NIR-emission wavelengths is based on the extension of the π-conjugation of the backbone of traditional fluorescent probes to lower the energy of the intramolecular charge transfer, but most of the dyes obtained by this strategy are quite large, causing a significant fading of images [6]. Furthermore, the noise background remains a disadvantage that limits the use of such NIR dyes. For their practical applications in FI, some photophysical factors, such as long emission wavelengths, high fluorescence quantum yields and large Stokes shifts, are necessary to avoid autofluorescence [13]. In this regard, the use of two different dyes that constitute an energy transfer pair or cassette, with close proximity and significant spectral overlap, is a promising strategy for tuning the emission color and enhancing the quantum efficiency of NIR dyes [14]. These bichromophoric systems allow for a high difference between the excitation and the emission energy, so suppressing photon scattering and diminishing tissue autofluorescence and have found application for the dynamic and high-contrast exploration of several pathological states. Following our research in the development of new bichromophoric systems for FI [15,16], in this paper, we describe the synthesis of a new energy donor–acceptor dyad **CB-Green** (Scheme 1), constituted of **Curc** and BODIPY **4**, that emits efficiently in the NIR region offering significant performances, both in terms of the ability to penetrate inside the cells and in terms of luminescent properties. BODIPY **2** itself has been proposed several times by us in bichromophoric systems as a significant red-emitting acceptor. On the other hand, curcumin has been previously used as a natural green-emitting donor [17] and the past results predicted that the dyad would be characterized by an efficient photoinduced energy transfer, active within the bichromophoric species. To investigate the use of a biocompatible nano carrier that could limit the drawbacks of the bichromophoric system due to its high lipophilicity, such as aggregation and shortening of emission time, and act as a “container” of both the new NIR dye and a drug, for a future theragnostic application, we decided to study the encapsulation of **CB-Green** in MCM-41 nanoparticles. These are silica particles with an average size between 50 and 80 nm and meso-structured pores with a 2D hexagonal arrangement. They exhibit singular features, such as ordered pore networks, with a very narrow and tunable pore size distribution (2–10 nm), a silanol-containing surface easily editable with different organic groups, large pore volumes (0.6–1 mL/g) and high surface areas (600–1000 m^2^/g) [18]. Their textural properties have inspired the idea of introducing drugs into the pore channels to be then locally released where needed, in drug delivery systems. Encapsulation into the porous silica structure also protects the guest molecules from degradation, while the silica surface offers several functionalization routes, enabling fine-tuning of the particles’ affinity towards specific environments and improving stability and uptake [19]. Furthermore, MCM-41 nanoparticles did not show reduction in cell viability, indicating their biocompatibility and bio-inertness and making them able to deliver organic molecules even in aqueous-based formulation, avoiding organic solvent toxicity.

## 2. Results and Discussion

### 2.1. Synthesis of Dyad CB-Green

The synthetic approach described in the upper part of Scheme 1 to reach the bichromophoric system **CB-Green**, and already used by authors [17], did not work with BODIPY **2** [20]. The functionalized curcumin **1** [21] was involved in a Sonogashira cross-coupling with BODIPY **2** without significant results. Pd catalyst, base and conditions were changed, and the reaction was even tried in the copper-free conditions, but the dyad **CB-Green** was obtained in traces, after difficult work-up. Therefore, it was necessary to move towards an alternative synthetic strategy, described in the lower part of Scheme 1. Starting from BODIPY **2**, it was reacted with propargyl alcohol, in a classical Sonogashira reaction, to obtain BODIPY **3** in 90% yield. BODIPY **3** was then converted into BODIPY **4** (Scheme 1) by PBr_3_ in absolute toluene, with 74% yield. The bichromophoric system **CB-Green** was obtained as a unique compound, lacking even traces of the bis-substituted BODIPY **4**/curcumin which could be isolated, from the reaction of **Curc** with BODIPY **4** in the presence of a slight excess of K_2_CO_3_, to avoid sensible degradation of the natural polyphenol. However, yields did not exceed the 54% in compound **CB-Green**, after purification.

### 2.2. Photochemical Characterization of Dyad CB-Green

**Figure 1:**
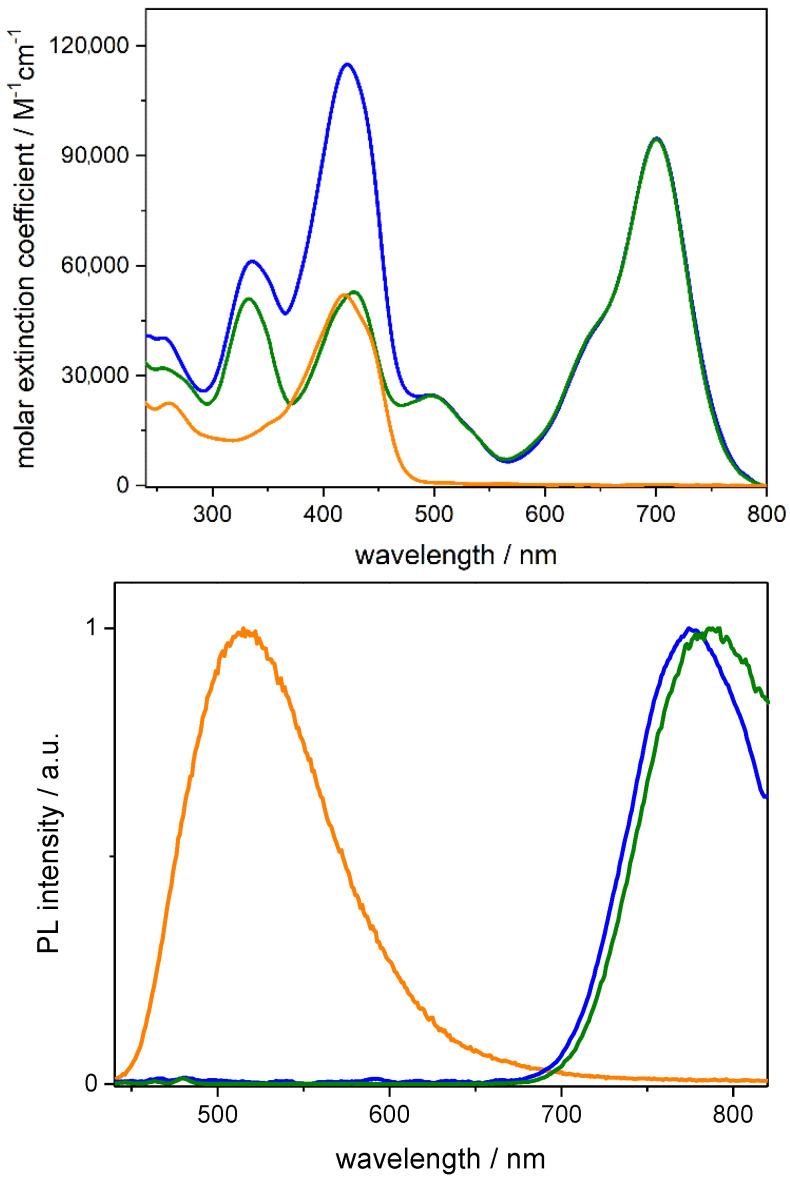
UV-Visible absorption (**top**) and emission (**bottom**) spectra of **Curc** (orange), **4** (green) and **CB-Green** (blue), recorded from micromolar acetonitrile solutions; emission was measured at λ_exc_ = 420 nm. Please note that the absorption maxima at lower energy lies at 418 nm for **Curc** and at 705 nm for **4** and **CB-Green**.

The absorption and emission spectra of the free compounds **Curc**, BODIPY **4** and **CB-Green** in solution are reported in Figure 1 and all the photophysical data are summarized in Table 1.

**Figure 2:**
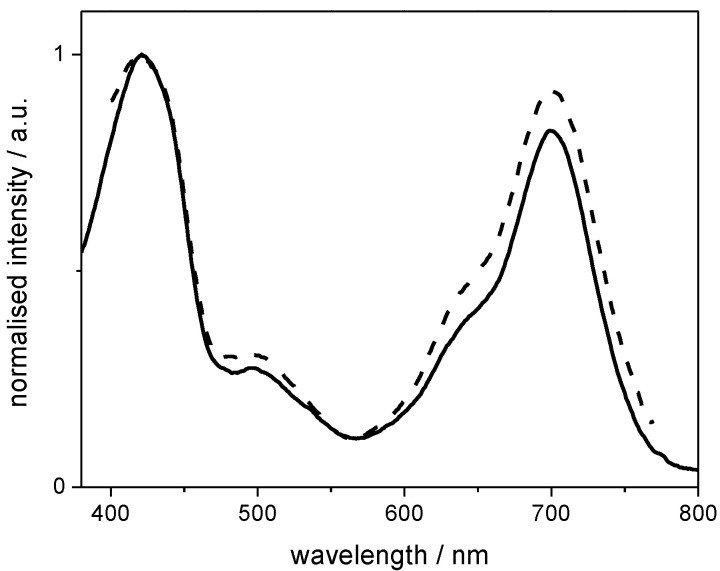
Comparison between the absorption spectrum (solid line) and the excitation spectrum (dashed line) of **CB-Green**; the latter was recorded at λ_em_ = 790 nm. Note that the intensity ratio between the maxima at 410 nm and 705 nm remained approximately constant between the absorption and excitation spectra.

From the absorption spectra, it is possible to distinguish the contributions of the two chromophores. The absorption band centered at around 420 nm is assigned to the **Curc** moiety, whereas the lowest-energy absorption band around 700 nm is centered on the BODIPY subunit and it is attributed to a π→π* transition with a partial charge transfer character, in which the lone pairs of the amine groups are involved. The emission spectrum of **CB-Green** (blue line) is dominated by a fluorescence band with a maximum at about 770 nm that is identical to the one exhibited by BODIPY **4** (green line). This, and the absence of any residual emission from the donor subunit, indicate an almost quantitative energy transfer from the higher energy absorber **Curc** to the lower-lying excited state centered on the BODIPY fragment. This is also confirmed by the comparison of the excitation spectrum of **CB-Green**, registered at 790 nm, with the absorption one (Figure 2).

### 2.3. Synthesis of Dye-Loaded Silica Nanoparticles

A specific type of mesoporous silica nanoparticles (MSNs) was chosen, namely the MCM-41, due to the presence of an ordered hexagonal array of pores, with mean size of 3–4 nm, where guest dyes can be encapsulated, and also due to the relatively small particle size, 70–100 nm, which is suitable for cell internalization [22]. The MSNs were doped with the bichromophoric compound **CB-Green** and the subunits, **Curc** and **4**, respectively, with the objective of studying the spectroscopic and biologic behavior of the dyes inside the nanoparticles and to determine possible variations in the energy transfer process. The experimental approach that was used for the encapsulation of the dyes took advantage of the well-established soft-template route used for all types of mesoporous silica materials. A micellar template was made by using an amphiphilic surfactant that is able to self-assemble in water and form rod-like micelles with a hexagonal arrangement. For MCM-41 particles, the surfactant of choice was cetyl trimethylammonium bromide (CTAB). After the first step, a suitable silica precursor was added, which hydrolyses and polymerizes around the template, forming the final silica network [22,23]. The precursor was tetraethyl orthosilicate (TEOS), the hydrolysis of which is facilitated in a basic aqueous solution, using 2 M NaOH. The dyes were added during the first step, in which, due to their insolubility in water, they spontaneously diffused through the CTAB micelles and entered the solvent-free, rigidified, hydrophobic core. To enable a homogeneous dispersion of the dyes, minimize intermolecular aggregation and quenching effects, prevent structural disruption of the micelles and, consequently, of the silica particles, we used a low dye loading, at a 99:1 molar ratio of CTAB:dye for each of the host–guest systems, hereafter named **Curc@MSN**, **4@MSN** and **CB-Green@MSN**. Such low content of dye was chosen on the basis of previous experiments showing that high loading of guest BODIPY species inside the nanoparticle resulted in fluorescence quenching [24,25]. Despite the relatively high temperature (80 °C) and pH (~12) used in the silica synthesis, the dyes showed excellent chemical stability and underwent no degradation.

### 2.4. Photochemical Characterization of the Dye-Loaded Silica Nanoparticles

**Figure 3:**
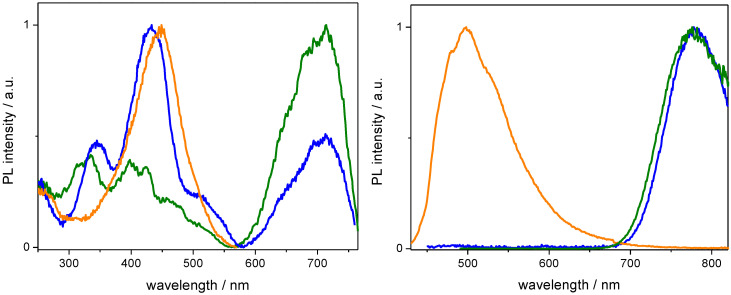
Normalized excitation (**left**) and emission (**right**) spectra of **Curc@MSN** (orange), **4@MSN** (green) and **CB-Green@MSN** (blue), recorded from 0.3 mg/mL cyclohexane suspensions at λ_em_ = 780 nm and λ_exc_ = 420 nm, respectively.

The dye-loaded MSNs were characterized by UV-Visible absorption and luminescence spectroscopy, and their properties were compared to those of the free dyes, to study the effect on the energy transfer inside the nanoparticles. Optical measurements were performed from suspensions of the materials in a solvent where the particles could be finely dispersed, to limit the intense light scattering that is typically generated by the suspended particles; the best solvent for such a purpose turned out to be cyclohexane and 0.3 mg/mL suspensions were used for the measurements. However, the absorption spectra still showed pronounced light scattering; therefore, only the excitation spectra are reported. Figure 3 shows the excitation and emission spectra of **Curc@MSN**, **4@MSN** and **CB-Green@MSN** recorded from the suspensions.

The spectra of the dye-loaded MSNs had an analogous trend to that observed for the dyes in solution. The excitation spectra were essentially additive, whereas the emission spectra showed only the component of the acceptor subunit BODIPY. The time-resolved fluorescence measurements also confirmed the same trend shown in solution and no residual emission nor excited state lifetime from the curcumin unit was detected.

### 2.5. Biocompatibility and Cellular Uptake Evaluations

**Figure 4:**
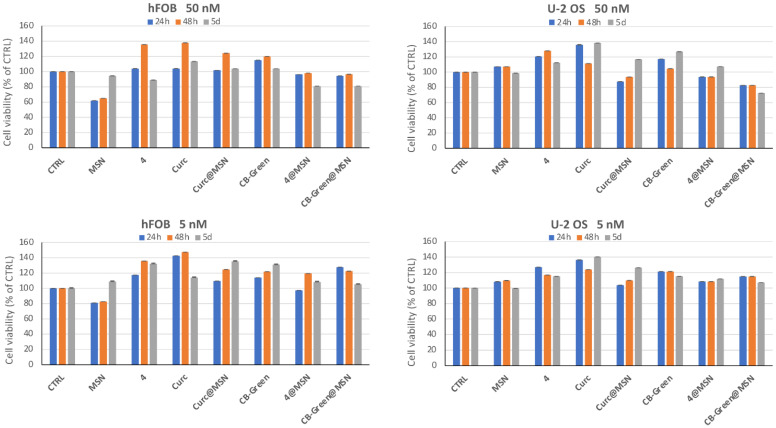
Cell viability test performed on hFOB and U-2 OS cells after 24 h, 48 h and 5 days of incubation with **MSN, 4, Curc, Curc@MSN, CB-Green, 4@MSN, CB-Green@MSN** molecules (50 and 5 nM). Data are represented as means ± SD of three independent experiments. Please note that the concentrations used refer to the amount of dye present in the nanostructure, as for MSN the concentration used was equal to 3.5 mg/mL for the series related to the 50 nM concentration of dyes and equal to 0.35 mg/mL for the 5 nM series (the amount of silica remained almost constant along the hybrid system series).

We performed the biological studies on two human osteoprogenitor cell lines, the human fetal osteoblastic cells (hFOB 1.19) and human bone osteosarcoma epithelial cells (U-2 OS). Both can be considered good models of normal and cancer bone cells, respectively. We initially investigated the cytotoxicity of the free molecules **Curc**, compound **4** and **CB-Green**, and the loaded nanoparticles **Curc@MSN**, **4@MSN** and **CB-Green@MSN**. In particular, the biocompatibility of such compounds was tested by MTS cell viability assay and the results confirmed that free molecules and hybrid systems at concentrations of 50 and 5 nM did not cause any evident trace of cellular suffering even after five days from administration, thus, underlining that they are safe enough to be used for imaging purposes. (Figure 4) Data were collected for other cell lines and are shown in supplementary material (see Table S1 in Supplementary Materials) and in all cases the non-cytotoxicity of the compounds under study was confirmed.

**Figure 5:**
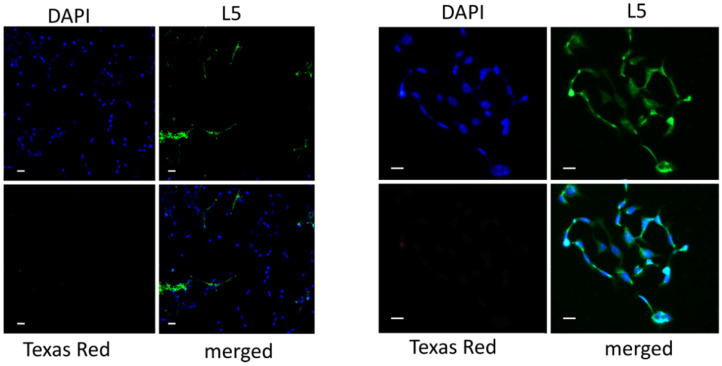
Fluorescence microscope images of hFOB 1.19 incubated with **Curc** (**left**) and with **Curc@MSN** (**right**). Bar = 1 μm.

To investigate cellular internalization and localization of **Green** and **CB-Green@MSN** and to compare the results with those obtained for **Curc**, BODIPY **4** and their corresponding MCM-41 systems, we performed cell labeling and fluorescence microscopy. The results regarding **Curc** and **Curc@MSN** are illustrated in Figure 5. Due to its lipophilicity, **Curc** dispersed in the aqueous solvent failed to penetrate the cell membrane, remaining as luminescent green crystals on the outside of the cells, (left panel of Figure 5) while its hybrid analogous spread within the cytoplasm with a green light, (right panel), showing how encapsulation in MCM-41 led to permeabilization, and supporting the ability of the MSNs to cross the plasmatic membrane without causing any damage to the cell.

**Figure 6:**
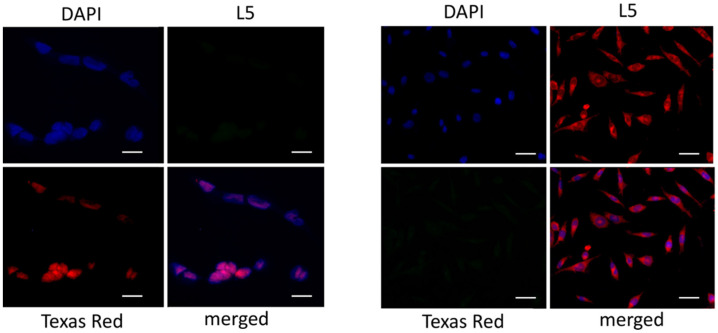
Fluorescence microscope images of hFOB 1.19 incubated with **4** (**left**) and with **4@MSN** (**right**). Bar = 1 μm.

Figure 6 illustrates BODIPY **4** and the MCM-41-loaded analogous **4@MSN**, both showing enlightening of red inside the cell but with two different localizations. The comparison between DAPI stain and **4** labeling of hFOB cells (left panel) clearly suggests that BODIPY **4** penetrated the nuclear membrane of cells and focused on the nucleus, which appeared illuminated with a bright red light. The red signals of hybrid **4@MSN**, instead, were mainly localized in the cytoplasm.

**Figure 7:**
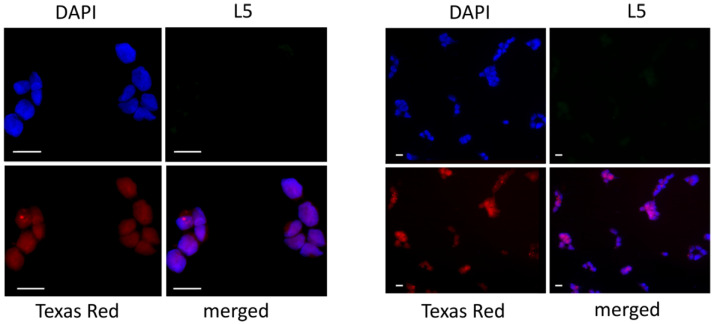
Fluorescence microscope images of hFOB.19 incubated with **CB-Green** (**left**) and with **CB-Green@MSN** (**right**). Bar = 1 μm.

Noteworthy, both **CB-Green** and **CB-Green@MSN** (Figure 7) illuminate the cellular environment with high efficiency even at concentrations of the 5 nM order, confirming that the low dosage of the dye and its hybrid system could not interfere with cell viability. Our microscope filter (Texas red filter set) did not allow reaching the wavelengths at which the two systems emitted to the maximum (790 nm), although images appeared bright and well-defined, thus, indicating that **CB-Green** and **CB-Green@MSN** performed significantly as biocompatible NIR dyes. Finally, it is worth noting that **CB-Green** illuminated with a red emission, typical of its acceptor component, BODIPY **4**, mainly in the nuclei of hFOB cells, (left Panel), while **CB-Green@MSN** was localized in the cytoplasm and, in particular, in the perinuclear region.(right Panel) The similar localization observed for **Curc@MSN** and **4@MSN** suggests that dimensions of MCM-41 nanoparticles prevented the penetration of the nuclear membrane, on one hand, offering the possibility of differentiating the localization of the light with respect to **CB-Green** within the cellular compartments, on the other hand.

## 3. Material and Methods

### 3.1. Chemicals

Solvents were purified according to standard procedures. All the syntheses were monitored by TLC on commercially available precoated plates (silica gel 60 F254), and the products were visualized with vanillin [1 g dissolved in MeOH (60 mL) and conc. H_2_SO_4_ (0.6 mL)] and UV lamp. Silica gel 60 was used for column chromatography.

### 3.2. Instrumentation

Proton (^1^H) and carbon (^13^C) NMR spectra were recorded on a Varian 500 spectrometer (at 500 MHz for ^1^H; and 125 MHz for ^13^C) using DMSO-*d_6_* as solvent. Chemical shifts were given in parts per million (ppm) (δ relative to residual solvent peak for ^1^H and ^13^C), coupling constants (J) were given in Hertz, and the attributions were supported by heteronuclear single-quantum coherence (HSQC) and correlation spectroscopy (COSY) experiments. Chemical shifts were reported in ppm relative to DMSO*-d_6_* (2.49 ppm). Numbering of Carbon atoms of compounds **3**, **4** and **CB-Green** are shown in Scheme 1. Combustion analyses were carried out with a FISONS EA1108 elemental analyzer. UV/Vis absorption spectra were taken on a Jasco V-560 spectrophotometer. For steady-state luminescence measurements, a Jobin Yvon-Spex Fluoromax 2 spectrofluorimeter was used, equipped with a Hamamatsu R3896 photomultiplier. The spectra were corrected for photomultiplier response using a program purchased with the fluorimeter. For the luminescence lifetimes, an Edinburgh OB 900 time-correlated single-photon-counting spectrometer was used. As excitation sources, a Hamamatsu PLP 2 laser diode (59 ps pulse width at 408 nm) and/or the nitrogen discharge (pulse width 2 ns at 337 nm) was employed. Emission quantum yields for de-aerated solutions were determined using the optically diluted method. As luminescence quantum yield standards, we used a trimethylammonium-phenylstyryl BODIPY species (ϕ = 0.69 in ACN). Microscopy images were acquired using a Leica DM5000B microscope equipped with DAPI, L5 and Texas Red fluorescence filters, using Leica LAS X acquisition software.

Experimental uncertainties were as follows: elemental analysis, 0.04%; absorption maxima, ±2 nm; molar absorption coefficient, 10%.

### 3.3. Synthesis

**Compound 3.** [Pd(PPh_3_)_2_Cl_2_] (0.0112 mmol) was added to a degassed solution of boron difluoro-4-(1E)-2-[5-[[5-[(1E)-2-[4-(dimethylamino) phenyl] ethenyl] -3-methyl-2*H*-pyrrol-2-ylidene-](4-iodophenyl methyl]-4-methyl -1*H*-pyrrol-2-yl-]ethenyl]-N,N-dimethylbenzenaminate (**2**) [20] (400 mg, 0.56 mmol) and propargyl alcohol (40 μL, 38 mg, 0.67 mmol) in DMF/TEA (12 mL, 5:1). In a sealed tube, the mixture was heated, at 80 °C, under argon, for 4 h, until the disappearance of **2** was observed by TLC (CHCl_3_/Hexane 90:10). Solvents were removed under reduced pressure. The reaction crude was dissolved in DCM and then filtered over celite/silica 1:2. The solution was evaporated to dryness to obtain **3** as a deep green solid in 90% yield, without needing any purification. *Rf*: 0.60 (CHCl_3_/Hexane 90:10^1^H NMR (DMSO-*d_6_*): *δ* 7.61–7.58 (2H, d, *J* = 7.8, 2× H-5), 7.48–7.40 (8H, m, 2× H-6, 2× H-14, 4× H-18), 7.31 (2H, part B of an AB system, *J* = 16, 2× H-15), 6.88 (2H, s, 2× H-11), 6.79 (4H, d, *J* = 8.8, 4× H-17), 5.37 (1H, t, *J* = 5.9 Hz, -OH), 4.34 (2H, d, *J* = 5.9 Hz, H_2_-1), 3.00 (12H, 4× -NC*H_3_*-20), 1.41 (6H, s, 2× C*H_3_*-13). ^13^C NMR: *δ* 152.7 (C- q), 151.5 (C-q), 140.7 (Cq), 137.7 and 113.7 (2× C-14, 2× C-15), 135.1 (Cq), 132.4 (Cq), 132.3 (2× C-5), 129.6 (Cq), 129.2 (2× C-6, 4× C-18,), 124.3 (Cq), 123.6 (Cq), 118.2 (2× C-11), 112.6 (4× C-17), 91.6 and 83.6 (C-2, C-3), 49.9 (C-1), 40.2 (4× C-20), 14.7 (2× C-13). Anal. Calcd. for C_40_H_39_BF_2_N_4_O (640,57) C, 75.00; H, 6.14; N, 8.75. Found: C, 74.93; H, 6.12; N, 8.75.

**Compound 4.** A solution of **3** (50 mg, 0.08 mmol) in abs. toluene (5 mL) under argon was cooled to 0 °C in an ice-bath. PBr_3_ (31 μL, 70 mg, 0.26 mmol, 3.3 eq) was added dropwise and the reaction mixture was stirred at 0 °C for 10′ and then it was allowed to warm to rt. The disappearance of **3** was followed by TLC (CHCl_3_/Hexane 90:10). After 6 h saturated NaCO_3_ solution was added. The phases were separated, and the organic layers were washed with water and brine, dried over MgSO_4_, filtered and concentrated under reduced pressure. The reaction crude was purified by silica gel column chromatography (eluants: CHCl_3_/Hexane 90:10) to obtain **4** as a deeply green solid, yield 74%. *Rf*: 0.95 (CHCl_3_). ^1^H NMR (DMSO-*d_6_*): *δ* 7.64–7.61 (2H, d, *J* = 7.9, 2× H-5), 7.47–7.40 (8H, m, 2× H-6, 2× H-14, 4× H-18,), 7.29 (2H, part B of an AB system, *J* = 15.7, 2× H-15), 6.87 (2H, s, 2× H-11), 6.78 (4H, d, *J* = 8.3, 4× H-17), 4.54 (2H, s, H_2_-1), 3.00 (4× -NC*H_3_*-20), 1.39 (6H, s, 2× C*H_3_*-13). Anal. Calcd. for C_40_H_38_BBrF_2_N_4_ (703,47): C, 68.29; H, 5.44; N, 7.96. Found: C, 68.49; H, 5.43; N, 7.95.

**CB-Green.** To a solution of **Curc** (30 mg, 0.08 mmol) in dry acetone (10 mL), **4** (75 mg, 0.106 mmol) and K_2_CO_3_ (22 mg, 0.16 mmol) were added. The mixture was stirred at reflux temperature for 48 h. The reaction was monitored using TLC (CHCl_3_/MeCN 99.5:0.5) following the disappearance of **4**. The solvent was removed under reduced pressure. The crude was purified by column chromatography (eluants: CHCl_3_ up to CHCl_3_/MeCN 99.5:0.5) on silica gel. The column afforded compound **CB-Green** as a green solid, 54% yield. *Rf*: 0.50 (CHCl_3_/MeCN 99.5:0.5). ^1^H NMR (DMSO-*d_6_*): *δ* 7.65–7.15 and 6.89–6.75 (28H, two m, H-1, H-3, H-4, H-8, H-9, H-13, H-14, H-16, H-19, H-20, 2× H-26, 2× H-27, 2× H-32, 2× H-35, 2× H-36, 4× H-38, 4× H-39), 6.1 (1H, s, H-11), 5.15 (2H, s, H_2_-22), 3.87 and 3.83 (6H, two s, 2× [-OCH_3_]), 3.00 (12H, 4× H-41), 1.4 (6H, s, 2× C*H_3_*-34). ^13^C NMR: δ 184.3 and 182.9 (C-10, C-12), 152.7 (Cq), 151.4 (Cq), 149.8 (Cq), 149.0 (Cq), 148.4 (C-q), 141.5 (Cq), 140.7 (Cq), 137.7 and 111.8 (2× C-35, 2× C-36), 136.1(C-q), 135.0 (Cq), 132.6 (2× C-26), 132.3 (C-8, C-14), 129.8 (Cq), 129.2 (2× C-27, 4× H-39), 129.0 (Cq), 126.7, 124.4, 123,6, 123.0, 122.8 and 122.5 (C1, C-3, C-4, C-16, C-19, C-20), 121.5 (Cq), 118.2 (Cq), 117.5 (Cq) 116.1 (2× C-32), 114.1 (Cq), 113.6 (Cq), 112.6 (C-9, C-13, 4× C-38,), 111.3(Cq), 101.4 (C-11), 86.6 and 86.4 (C-23, C-24), 57.1 (C-22), 56.1 (C-7, C-21), 40.4 (4× C-41), 14.8 (2× C-34). Anal. Calcd. for C_61_H_57_BF_2_N_4_O_6_ (990,94): C, 73.94; H, 5.80; N, 5.65. Found: C, 74.15; H, 5.79; N, 5.67.

**Dye-loaded Silica Nanoparticles**. Cetyltrimethylammonium bromide (60.5 mg) was dissolved in 30 mL of water using an ultrasound bath. The dye was dissolved in a minimal amount of acetonitrile (500 μL) and transferred into the water solution of CTAB under stirring, after which a homogeneous colloidal suspension was formed. The dyes **4** (0.6 mg), **Curc** (0.31 mg) and **CB-Green** (0.83 mg) were added separately in 1:99 molar ratios in respect to CTAB. This was then followed by the addition of 2 M aqueous NaOH (0.22 mL, 0.44 mmol). The mixture was heated to 80 °C and the silica precursor tetraethylorthosilicate (283 mg, 0.3 mL, 1.37 mmol) was added, at which point silica particles started to form quickly. The suspension was kept stirring at reflux for 2 h, then stopped and allowed to cool down to room temperature. The mixture was filtered under vacuum, washed with deionized water and finally dried in a vacuum desiccator. All powders fluoresced strongly under a UV lamp.

### 3.4. Cell Culture and Cell Viability Assay

Human fetal osteoblastic cell line (hFOB 1.19) and human bone osteosarcoma epithelial cells (U-2 OS) were obtained from the American Type Culture Collection (ATCC, Manassas, VA, United States). HFOB 1.19 were cultured in 1:1 mixture of Ham’s F12 Medium -Dulbecco’s Modified Eagle’s Medium (Merk Life Science S.r.l., Milan, Italy), supplemented with 2.5 mM L-glutamine (L-glu, Merk Life Science S.r.l., Milan, Italy), 0.3 mg/mL G418 (ThermoFisher, Waltham, MA USA); 10% Fetal Bovine Serum (FBS, Merk Life Science S.r.l., Milan, Italy) and 1% penicillin/streptomycin/amphotericin (PSA, Merk Life Science S.r.l., Milan, Italy). U-2 OS were grown in McCoy’s 5a Medium Modified (Merk Life Science S.r.l., Milan, Italy) supplemented with 2.5 mM L-glu, 10% FBS and 1% PSA. Both cell lines were incubated in a humidified atmosphere containing 5% CO_2_ at 37 °C. The medium was replaced twice a week and cells were split at about 80% of confluence. Cell viability assay was performed by MTS (3-(4,5-dimethylthiazol-2-yl)-5-(3-carboxymethoxyphenyl)-2-(4-sulfophenyl)-2H-tetrazolium) (Cell Titer96^®^ Aqueous One Solution Proliferation Assay Kit, Promega, Madison, WI, USA) according to the manufacturing protocol. For MTS assay, 5 × 10^3^ cells were cultured in a 96-well plate, with specific medium and incubated in a humidified atmosphere containing 5% CO_2_ at 37 °C for 24 h. Then, compounds and hybrid systems under study were added separately, at two different concentrations (5 and 50 nM) and cells re-incubated for 24 h, 48 h and 5 days. After 24 h, 48 h and 5 days, MTS reagent was added to the culture medium and the plate was incubated for 1 h at 37 °C. Finally, the plate was shacked shortly and the absorbance at 490 nm measured using a synergy HT plate reader (BioTek Instruments, Inc., Winooski, VT, USA). Each biological system compound was analyzed in triplicate for each concentration and time. Data were reported as percentage of the control ± standard deviation.

The HL-60 cells were obtained from ATCC^®^ (CCL-240, Rockville, MD, USA), while their variant, HL-60R, were derived by exposure to gradually increasing concentrations of doxorubicin. The molecular characterization of HL-60R cells was carried out previously [26] The human breast cancer cell lines MDA-MB-231 and the colorectal adenocarcinoma cell lines Caco-2 were obtained from ATCC^®^ (respectively HTB-26™ and HTB-37™—Rockville, MD, USA). The HL-60 and HL-60R cells were routinely maintained in Roswell Park Memorial Institute (RPMI) 1640 (HyClone Europe Ltd., Cramlington UK), while MDA-MB-231 cells were cultured in Dulbecco’s Modified Eagle Medium (DMEM) (HyClone Europe Ltd., Cramlington, UK) supplemented with 10% heat inactivated fetal calf serum, 2 mM L-glutamine, 100 units/mL penicillin and 100 µg/mL streptomycin (all reagents were from HyClone Europe Ltd., Cramlington, UK) in a humidified atmosphere at 37 °C in 5% CO_2_. Cells with a narrow range of passage numbers were used for all experiments. The cultures were routinely tested for Mycoplasma infection.

Cells were seeded on 96-well plates at a density of 5000 cells/well and incubated overnight at 37 °C. After 24 h, at time 0 the medium was replaced with a fresh complete medium supplemented with the investigated systems. After 72 h of treatment, 15 µL of Promega Corp. commercial solution (Madison, WI, USA) containing 3-(4,5-dimethylthiazol-2-yl)-5-(3-carboxymethoxyphenyl)-2-(4-sulfophenyl)-2H-tetrazolium (MTS) and phenazine ethosulfate was added to each well and the plates were incubated at 37 °C at 5% CO_2_ for 2 h. Using a microplate reader (iMark Microplate Reader; Bio-Rad Laboratories, Inc., Hercules, CA, USA), the bio-reduction of the MTS dye was evaluated by measuring the absorbance of each well at 490 nm. Cytotoxicity was expressed as a percentage of measured absorbance relative to that of control cells. Data were expressed as mean ± standard error (S.E.) of at least three different experiments performed in duplicate.

## 4. Conclusions

We have described the synthesis of a novel dyad in which the donor curcumin is bonded to an acceptor BODIPY to yield a bichromophoric system. **CB-Green** possesses all the desirable properties of a bioimaging probe: large Stokes shift, long emission wavelengths, high fluorescence quantum yield, and biocompatibility. As much as the conjugation of curcumin to BODIPY results in the loss of some of the inherent advantages of this subunit (cytotoxicity, antitumor activity, and so on), the new system allows the biological material to be illuminated in the near-IR by exciting it in the blue as well. This behavior is particularly interesting from an application point of view because the spectral emission range of the new species lies within the biological window.

In addition, it presents a specific intracellular staining. **CB-Green** targets the nucleus of the studied cell lines, thus supporting the idea that small-size dye molecules are desirable tools in FI since they enter the cell fast, locate in the target region and, in this specific case, do not induce any stress to cells up to 5 days. Encapsulation of **CB-Green** and the free components of the bichromophoric system, **Curc** and BODIPY **4,** inside MCM-41 silica nanoparticles allows for all three compounds to mask their lipophilic nature, overpass the cellular membrane and locate within the cytoplasm without losing brightness. The dyad maintains a very efficient energy transfer even within the hybrid silica system, which confirms the viability of the encapsulation strategy for imaging purposes.

## Figures and Tables

**Table 1:** Spectroscopic data of the bichromophoric **CB-Green** species and the subunits **Curc** and **4**, recorded from acetonitrile solutions.

Compound	Absorption	Emission		
	λ_max_, nm (ε, M^−1^ cm^−1^)	λ_max_, nm	τ, ns	Φ
**CB-Green**	705 (95,000)	770	2.2	0.18
**Curc**	418 (52,900)	520	0.66	0.1
**4**	423 (116,100) 705 (94,800)	770	2.2	0.2

## Data Availability

Not applicable.

## Supplementary Materials

The following supporting information can be downloaded at: https://www.mdpi.com/article/10.3390/ijms23179542/s1.
