# Supplementary material for: A Curcumin-BODIPY Dyad and Its Silica Hybrid as NIR Bioimaging Probes
Source: Int J Mol Sci. 2022 Aug 23;23(17):9542. doi: 10.3390/ijms23179542 (PMC9455722; doi:10.3390/ijms23179542)
Supplement: Supplementary file 1 [file ijms-23-09542-s001.zip › ijms-1873306-supplementary.pdf]

A Curcumin-BODIPY dyad and its silica hybrid as NIR bioimaging probes

C.M.A. Gangemi,[a] T.M. G. Salerno,[a] A. Barattucci,[a] F. Cucinotta,[b]*P. Bonaccorsi,[a*] G. Calabrese,[a] P. Poma,[c] M. G. Rizzo[a] and F. Puntoriero[a]

[a]Dipartimento di Scienze Chimiche Biologiche Farmaceutiche ed Ambientali (CHIBIOFARAM), Università degli Studi di Messina, Messina, Italy

[b] School of Natural and Environmental Sciences, Newcastle University, Newcastle upon Tyne, United Kingdom

[c]Dipartimento di Scienze e Tecnologie Biologiche, Chimiche e Farmaceutiche (STEBICEF), Università degli Studi di Palermo, Palermo, Italy

Table of contents

	Page
**Materials and Methods**	……1
**Synthesis**	……3
**Biological study**	……4
**References**	……5
**NMR Spectra**	……6

Materials and Methods

Chemicals

Solvents were purified according to standard procedures. All the syntheses were monitored by TLC on commercially available precoated plates (silica gel 60 F254), and the products were visualized with vanillin [1 g dissolved in MeOH (60 mL) and conc. H_2SO_4 (0.6 mL)] and UV lamp. Silica gel 60 was used for column chromatography.

Instrumentation

Proton (^1H) and carbon (^{13}C) NMR spectra were recorded on a Varian 500 spectrometer (at 500 MHz for ^1H; and 125 MHz for ^{13}C) using DMSO-d_6 as solvent. Chemical shifts are given in parts per million (ppm) (δ relative to residual solvent peak for ^1H and ^{13}C), coupling constants (J) are given in Hertz, and the attributions are supported by heteronuclear single-quantum coherence (HSQC) and correlation spectroscopy (COSY) experiments. Chemical shifts are reported in ppm relative to DMSO-d_6 (2.49 ppm). Numbering of Carbon atoms of compounds **3**, **4** and **CB-Green** are shown in Scheme 1. UV/Vis absorption spectra were taken on a Jasco V-560 spectrophotometer. For steady-state luminescence measurements, a Jobin Yvon-Spex Fluoromax 2 spectrofluorimeter was used, equipped with a Hamamatsu R3896 photomultiplier. The spectra were corrected for photomultiplier response using a program purchased with the fluorimeter. For the luminescence lifetimes, an Edinburgh OB 900 time-correlated single-photon-counting spectrometer was used. As excitation sources, a Hamamatsu PLP 2 laser diode (59 ps pulse width at 408 nm) and/or the nitrogen discharge (pulse width 2 ns at 337 nm) were employed. Emission quantum yields for deaerated solutions were determined using the optically diluted method. As luminescence quantum yield standards, we used a trimethylammonium-phenylstyryl BODIPY species (ϕ=0.69 in ACN). Microscopy images were acquired using a Leica DM5000B microscope equipped with DAPI, L5 and Texas Red fluorescence filters, using Leica LAS X acquisition software.

Synthesis

Compound 3. [Pd(PPh_3)$_2$$Cl_2$] (0.0112 mmol) was added to a degassed solution of boron difluoro-4-(1E)-2-[5-[[5-[(1E)-2-[4-(dimethylamino) phenyl] ethenyl] -3-methyl-2*H*-pyrrol-2-ylidene-](4-iodophenyl methyl]-4-methyl -1*H*-pyrrol-2-yl-]ethenyl]-N,N-dimethylbenzenaminate (**2**)[1] (400 mg, 0.56 mmol) and propargyl alcohol (40 µl, 38 mg, 0.67 mmol) in DMF/TEA (12ml, 5:1). In a sealed tube, the mixture was heated, at 80°C, under argon, for 4 h, until the disappearance of **2** was observed by TLC ($CHCl_3$/Hexane 90:10). Solvents were removed under reduced pressure. The reaction crude was dissolved in DCM and then filtered over celite/silica 1:2. The solution was evaporated to dryness to obtain **3** as a deep green solid in 90 % yield, without needing any purification. *Rf*: 0.60 ($CHCl_3$/Hexane 90:10^1H NMR (DMSO-d_6): δ 7.61-7.58 (2H, d, *J*=7.8, 2x H-5), 7.48-7.40 (8H, m, 2x H-6, 2x H-14, 4x H-18), 7.31 (2H, part B of an AB system, *J*=16, 2x H-15), 6.88 (2H, s, 2x H-11), 6.79 (4H, d, *J*=8.8, 4x H-17), 5.37 (1H, t, *J*=5.9 Hz, -OH), 4.34 (2H, d, *J*=5.9 Hz, H_2-1), 3.00 (12H, 4x -NCH_3-20), 1.41 (6H, s, 2x CH_3-13). ^{13}C NMR: δ 152.7 (C- q), 151.5 (C-q), 140.7 (Cq), 137.7 and 113.7

(2x C-14, 2x C-15), 135.1 (Cq), 132.4 (Cq), 132.3 (2x C-5), 129.6 (Cq), 129.2 (2x C-6, 4x C-18,), 124.3 (Cq), 123.6 (Cq), 118.2 (2x C-11), 112.6 (4x C-17), 91.6 and 83.6 (C-2, C-3), 49.9 (C-1), 40.2 (4x C-20), 14.7 (2x C-13). Anal. Calcd. for $C_{40}H_{39}BF_2N_4O$ (640,57) C, 75.00; H, 6.14; N, 8.75. Found: C, 74.93; H, 6.12; N, 8.75.

Compound 4. A solution of **3** (50 mg, 0.08 mmol) in abs. toluene (5 ml) under argon was cooled to 0°C in an ice-bath. PBr_3 (31 µl, 70 mg, 0.26 mmol, 3.3 eq) was added dropwise and the reaction mixture was stirred at 0°C for 10' and then it was allowed to warm to rt. The disappearance of **3** was followed by TLC ($CHCl_3$/Hexane 90:10). After 6h saturated $NaCO_3$ solution was added. The phases were separated, and the organic layers were washed with water and brine, dried over $MgSO_4$, filtered and concentrated under reduced pressure. The reaction crude was purified by silica gel column chromatography (eluants: $CHCl_3$/Hexane 90:10) to obtain **4** as a deeply green solid, yield 74%. *Rf*: 0.95 ($CHCl_3$). ^{1}H NMR (DMSO-d_6): δ 7.64-7.61 (2H, d, J=7.9, 2x H-5), 7.47-7.40 (8H, m, 2x H-6, 2x H-14, 4x H-18,), 7.29 (2H, part B of an AB system, J=15.7, 2x H-15), 6.87 (2H, s, 2x H-11), 6.78 (4H, d, J=8.3, 4x H-17), 4.54 (2H, s, H_2-1), 3.00 (4x -NCH_3-20), 1.39 (6H, s, 2x CH_3-13). Anal. Calcd. for $C_{40}H_{38}BBrF_2N_4$ (703,47): C, 68.29; H, 5.44; N, 7.96. Found: C, 68.49; H, 5.43; N, 7.95.

CB-Green. To a solution of **Curc** (30 mg, 0.08 mmol) in dry acetone (10 mL), **4** (75mg, 0.106 mmol) and K_2CO_3 (22 mg, 0.16 mmol) were added. The mixture was stirred at reflux temperature for 48h. The reaction was monitored using TLC ($CHCl_3$/MeCN 99.5:0.5) following the disappearance of **4**. The solvent was removed under reduced pressure. The crude was purified by column chromatography (eluants: $CHCl_3$ up to $CHCl_3$/MeCN 99.5:0.5) on silica gel. The column afforded compound **CB-Green** as a green solid, 54% yield. *Rf*: 0.50 ($CHCl_3$/MeCN 99.5:0.5). ^{1}H NMR (DMSO-d_6): δ 7.65-7.15 and 6.89-6.75 (28H, two m, H-1, H-3, H-4, H-8, H-9, H-13, H-14, H-16, H-19, H-20, 2x H-26, 2x H-27, 2x H-32, 2x H-35, 2x H-36, 4x H-38, 4x H-39), 6.1 (1H, s, H-11), 5.15 (2H, s, H_2-22), 3.87 and 3.83 (6H, two s, 2x [-OCH_3]), 3.00 (12H, 4x H-41), 1.4 (6H, s, 2x CH_3-34). ^{13}C NMR: δ 184.3 and 182.9 (C-10, C-12), 152.7 (Cq), 151.4 (Cq), 149.8 (Cq), 149.0 (Cq), 148.4 (C-q), 141.5 (Cq), 140.7 (Cq), 137.7 and 111.8 (2x C-35, 2x C-36), 136.1(C-q), 135.0 (Cq), 132.6 (2x C-26), 132.3 (C-8, C-14), 129.8 (Cq), 129.2 (2x C-27, 4x H-39), 129.0 (Cq), 126.7, 124.4, 123,6, 123.0, 122.8 and 122.5 (C1, C-3, C-4, C-16, C-19, C-20), 121.5 (Cq), 118.2 (Cq), 117.5 (Cq) 116.1 (2x C-32), 114.1 (Cq), 113.6 (Cq), 112.6 (C-9, C-13, 4x C-38,), 111.3(Cq), 101.4 (C-11), 86.6 and 86.4 (C-23, C-24), 57.1 (C-22) , 56.1 (C-7, C-21), 40.4 (4x C-41), 14.8 (2x C-34). Anal. Calcd. for $C_{61}H_{57}BF_2N_4O_6$ (990,94): C, 73.94; H, 5.80; N, 5.65. Found: C, 74.15; H, 5.79; N, 5.67.

Dye-loaded Silica Nanoparticles. Cetyltrimethylammonium bromide (60.5 mg) was dissolved in 30 mL of water using an ultrasound bath. The dye was dissolved in a minimal amount of acetonitrile (500 µl) and transferred into the water solution of CTAB under stirring, after which a homogeneous colloidal suspension was formed. The dyes **4** (0.6 mg), **Curc** (0.31 mg) and **CB-Green** (0.83 mg) were added separately in 1:99 molar ratios in respect with CTAB. This was then followed by the addition of 2 M aqueous NaOH (0.22 mL, 0.44 mmol). The mixture was heated to 80 °C and the silica precursor tetraethylorthosilicate (283 mg, 0.3 mL, 1.37 mmol) was added, at which point silica particles started to form quickly. The suspension was

kept stirring at reflux for 2 hours, then stopped and allowed to cool down to room temperature. The mixture was filtered under vacuum, washed with deionised water and finally dried in a vacuum desiccator. All powders fluoresced strongly under a UV lamp.

Cell culture and cell viability assay

Human fetal osteoblastic cell line (hFOB 1.19) and human bone osteosarcoma epithelial cells (U-2 OS) were obtained from the American Type Culture Collection (ATCC, Manassas, VA, United States). h-fob 1.19 were cultured in 1:1 mixture of Ham's F12 Medium - Dulbecco's Modified Eagle's Medium (Merk Life Science S.r.l., Milan, Italy), supplemented with 2.5 mM L-glutamine (L-glu, Merk Life Science S.r.l., Milan, Italy), 0.3 mg/ml G418 (ThermoFisher, Waltham, MA USA); 10% Fetal Bovine Serum (FBS, Merk Life Science S.r.l., Milan, Italy) and 1% penicillin/streptomycin/amphotericin (PSA, Merk Life Science S.r.l., Milan, Italy). U-2 OS were grown in McCoy's 5a Medium Modified (Merk Life Science S.r.l., Milan, Italy) supplemented with 2.5 mM L-glu, 10% FBS and 1% PSA. Both cell lines were incubated in a humidified atmosphere containing 5% CO_2 at 37 °C. The medium was replaced twice a week and cells were split at about 80% of confluence. Cell viability assay was performed by MTS (3- (4,5-dimethylthiazol-2-yl) -5- (3-carboxymethoxyphenyl) -2- (4-sulfophenyl) -2H-tetrazolium) (Cell Titer96® AQueous One Solution Proliferation Assay Kit, Promega, Madison, WI, USA) according to the manufacturing protocol. For MTS assay, $5x10^3$ cells were cultured in 96-well plate, with specific medium and incubated in a humidified atmosphere containing 5% CO_2 at 37 °C for 24h. Then, compounds and hybrid systems under study were added separately, at two different concentrations (5 and 50 nM) and cells re-incubated for 24h, 48h and 5 days. After 24h, 48h and 5 days, MTS reagent was added to the culture medium and the plate was incubated for 1 hour at 37 °C. Finally, the plate was shacked shortly and the absorbance at 490 nm measured using a synergy HT plate reader (BioTek Instruments, Inc., VT, United States). Each biological system compound was analysed in triplicate for each concentration and time. Data are reported as percentage of the control ± standard deviation. The HL-60 cells were obtained from ATCC® (CCL-240, Rockville, MD, USA), while their variant, HL-60R, were derived by exposure to gradually increasing concentrations of doxorubicin. The molecular characterization of HL-60R cells was carried out previously [2]The human breast cancer cell lines MDA-MB-231 and the colorectal adenocarcinoma cell lines Caco-2 were obtained from ATCC® (respectively HTB-26™ and HTB-37™—Rockville, MD, USA). The HL-60 and HL-60R cells were routinely maintained in Roswell Park Memorial Institute (RPMI) 1640 (HyClone Europe Ltd., Cramlington UK), while MDA-MB-231 cells were cultured in Dulbecco’s Modified Eagle Medium (DMEM) (HyClone Europe Ltd., Cramlington, UK) supplemented with 10% heat inactivated fetal calf serum, 2 mM L-glutamine, 100 units /mL penicillin and 100 µg/mL streptomycin (all reagents were from HyClone Europe Ltd., Cramlington, UK) in a humidified atmosphere at 37 ◦C in 5% CO_2. Cells with a narrow range of passage numbers were used for all experiments. The cultures were routinely tested for Mycoplasma infection. Cells were seeded on 96-well plates at a density of 5000 cells/well and incubated overnight at 37 ◦C. After 24 h, at time 0 the medium was replaced with a fresh complete medium supplemented with the investigated systems. After 72 h of treatment, 15 µL of Promega Corp.

commercial solution (Madison, WI, USA) containing 3-(4,5-dimethylthiazol-2-yl)-5-(3-carboxymethoxyphenyl)-2-(4-sulfophenyl)-2H-tetrazolium (MTS) and phenazine ethosulfate was added to each well and the plates were incubated at 37 °C at 5% CO_2 for 2 h. Using a microplate reader (iMark Microplate Reader; Bio-Rad Laboratories, Inc., Hercules, CA, USA), the bioreduction of the MTS dye was evaluated by measuring the absorbance of each well at 490 nm. Cytotoxicity was expressed as a percentage of measured absorbance relative to that of control cells. Data were expressed as mean ± standard error (S.E.) of at least three different experiments performed in duplicate.

Table S1 *. IC_{50} values (mean ± SE) in the cell lines*

	**HL-60**	**HL-60R**	**MDA-MB-231**	**Caco-2**
**4@MSN**	0.625 µM ± 0.18	52.0 µM ± 1.99	29.7 µM ± 9.0	60.2 µM ± 7.2
**Curc@MSN**	4.5 µM ± 1.9	68.0 µM ± 22.0	29.0 µM ± 13.4	41.2 µM ± 4.4
**CB-Green@MSN**	2.02 µM ± 1.3	49.0 µM ± 7.9	9.0 µM ± 2.8	33.5 µM ± 6.0
**CB-Green**	> 100 µM	> 100 µM	> 100 µM	> 100 µM
**MSN**	92.5 µM ± 42.5	> 100 µM	> 100 µM	> 100 µM

HL-60: human promyelocytic leukemia; **HL-60 R:** RA-resistant subclone of HL-60; **MDA-MB 231**: human breast adenocarcinoma; **Caco-2:** human colorectal adenocarcinoma.

References

[1] Bonaccorsi, P.; Papalia, T.; Barattucci, A.; Salerno, T.M.G.; Rosano, C.; Castagnola, P.; Viale, M.; Monticone, M.; Campagna, S.; Puntoriero, F. Localization-controlled two-color luminescence imaging via environmental modulation of energy transfer in a multichromophoric species. *Dalton Trans.* **2018**, *47*, 4733-4738.

[2] Notarbartolo, M.; Cervello, M.; Poma, P.; Dusonchet, L.; Meli, M.; D'Alessandro, N. Expression of the IAPs in multidrug resistant tumor cells. *Oncol. Rep.* **2004**, *11*, 133–136.

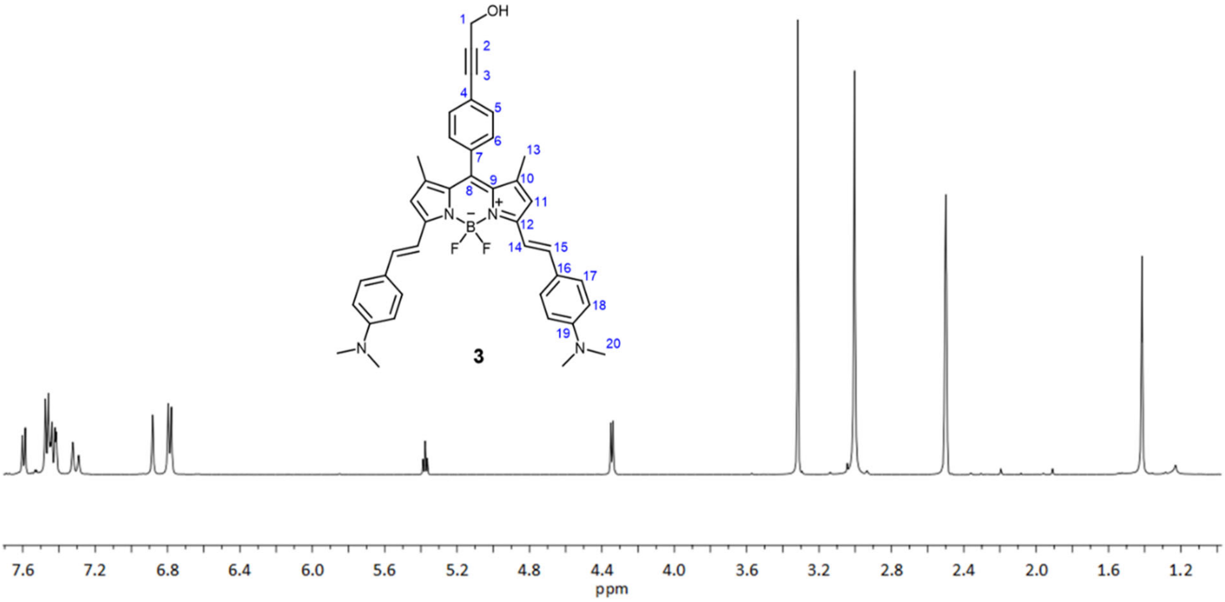

Figure S1. ^{1}H-NMR of compound **3** in DMSO-d_6

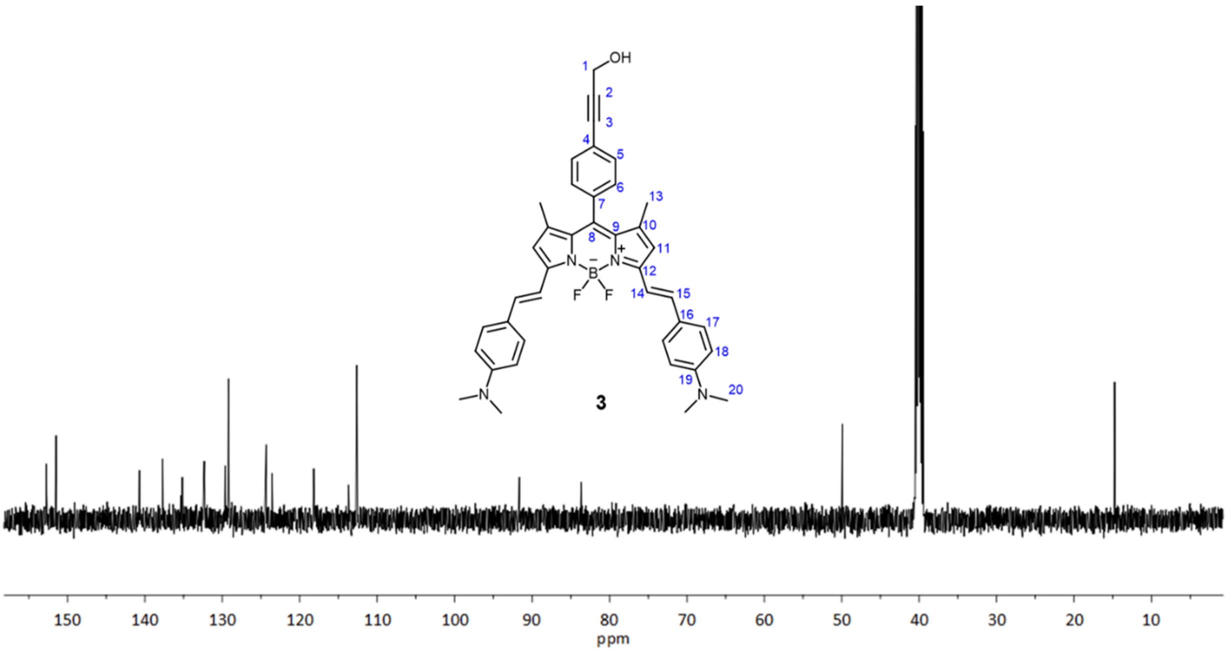

Figure S2. ^{13}C-NMR of compound **3** in DMSO-d_6

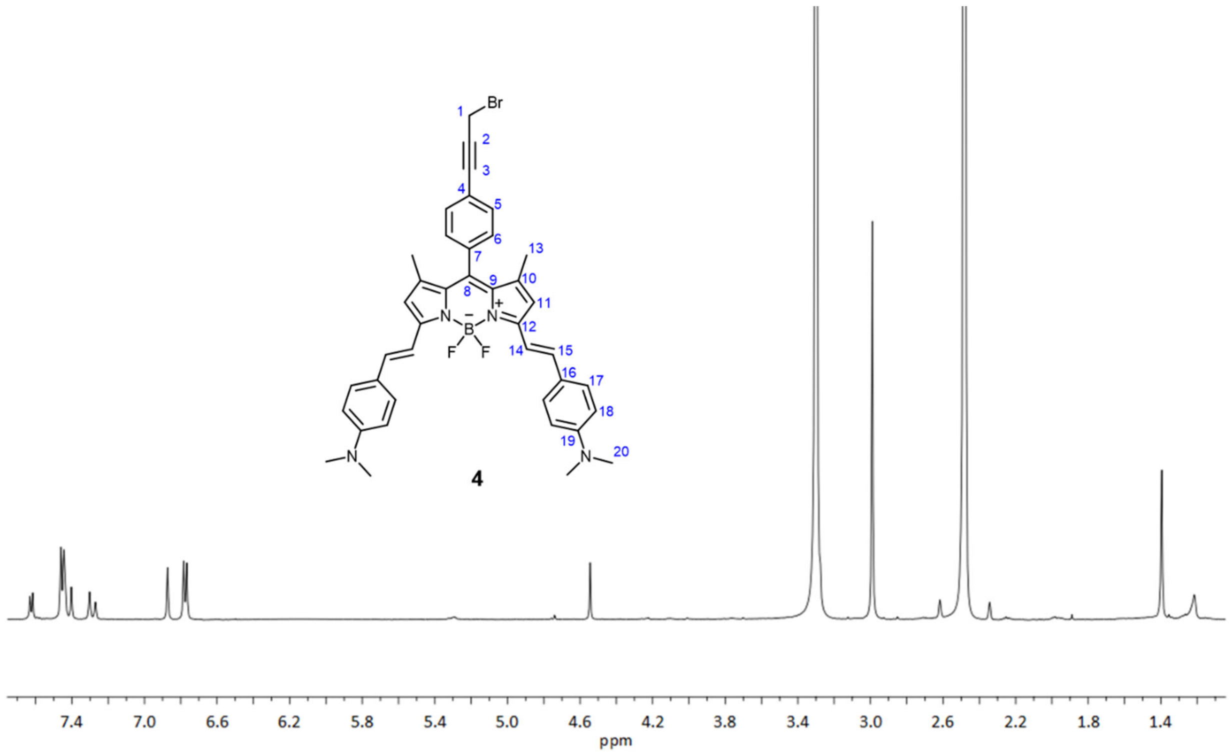

Figure S3. ^{1}H-NMR of compound **4** in DMSO-d_6

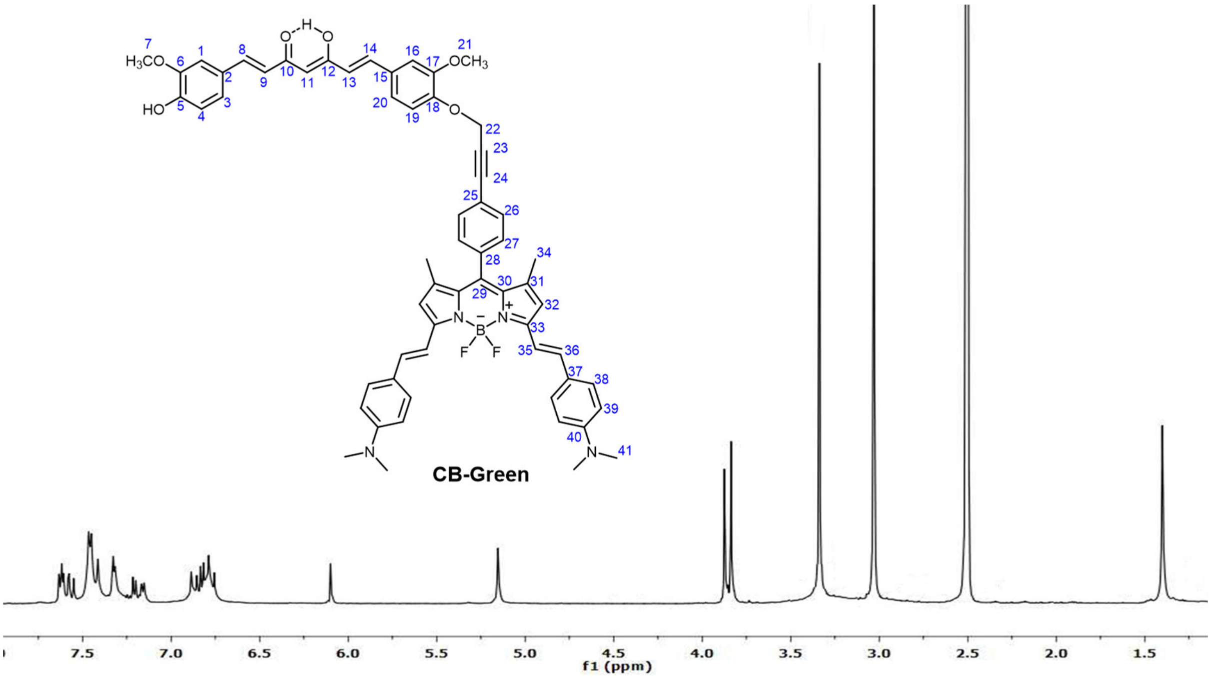

Figure S4. ^{1}H-NMR of compound **CB-Green** in DMSO-d_6

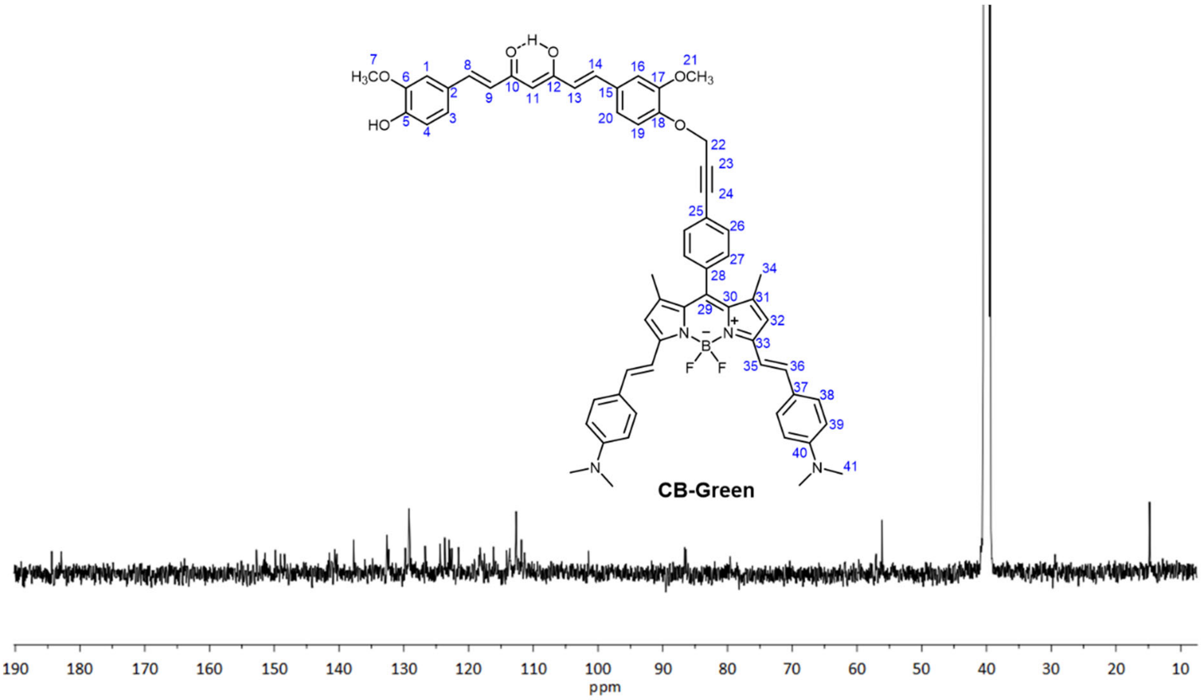

Figure S5. ^{13}C-NMR of compound **CB-Green** in DMSO-d_6
